# MicroRNA-195-5p Downregulation Inhibits Endothelial Mesenchymal Transition and Myocardial Fibrosis in Diabetic Cardiomyopathy by Targeting Smad7 and Inhibiting Transforming Growth Factor Beta 1-Smads-Snail Pathway

**DOI:** 10.3389/fphys.2021.709123

**Published:** 2021-09-30

**Authors:** Huaisheng Ding, Jianhui Yao, Hongxiang Xie, Chengyu Wang, Jing Chen, Kaiyong Wei, Yangyang Ji, Lihong Liu

**Affiliations:** Cardiovascular Department, Meishan People’s Hospital, Meishan, China

**Keywords:** diabetic cardiomyopathy, miR-195-5p, TGF-β1-smads-snail pathway, smad7, endothelial mesenchymal transition, myocardial fibrosis

## Abstract

Diabetic cardiomyopathy (DCM) is a complication of diabetes mellitus, which is associated with fibrosis and microRNAs (miRs). This study estimated the mechanism of miR-195-5p in endothelial mesenchymal transition (EndMT) and myocardial fibrosis in DCM. After the establishment of DCM rat models, miR-195-5p was silenced by miR-195-5p antagomir. The cardiac function-related indexes diastolic left ventricular anterior wall (LVAW, d), systolic LVAW (d), diastolic left ventricular posterior wall (LVPW, d), systolic LVPW (d), left ventricular ejection fraction (LVEF), and fractional shortening (FS) were measured and miR-195-5p expression in myocardial tissue was detected. Myocardial fibrosis, collagen deposition, and levels of fibrosis markers were detected. Human umbilical vein endothelial cells (HUVECs) were exposed to high glucose (HG) and miR-195-5p was silenced. The levels of fibrosis proteins, endothelial markers, fibrosis markers, EndMT markers, and transforming growth factor beta 1 (TGF-β1)/Smads pathway-related proteins were measured in HUVECs. The interaction between miR-195-5p and Smad7 was verified. *In vivo*, miR-195-5p was highly expressed in the myocardium of DCM rats. Diastolic and systolic LVAW, diastolic and systolic LVPW were increased and LVEF and FS were decreased. Inhibition of miR-195-5p reduced cardiac dysfunction, myocardial fibrosis, collagen deposition, and EndMT, promoted CD31 and VE-cadehrin expressions, and inhibited α-SMA and vimentin expressions. *In vitro*, HG-induced high expression of miR-195-5p and the expression changes of endothelial markers CD31, VE-cadehrin and fibrosis markers α-SMA and vimentin were consistent with those *in vivo* after silencing miR-195-5p. In mechanism, miR-195-5p downregulation blocked EndMT by inhibiting TGF-β1-smads pathway. Smad7 was the direct target of miR-195-5p and silencing miR-195-5p inhibited EndMT by promoting Smad7 expression. Collectively, silencing miR-195-5p inhibits TGF-β1-smads-snail pathway by targeting Smad7, thus inhibiting EndMT and alleviating myocardial fibrosis in DCM.

## Introduction

Diabetes is characterized by dynamic changes in blood glucose levels, which are associated with multiple complications, especially cardiomyopathy and endothelial dysfunction (Zhang and Sun, 2020). Diabetic cardiomyopathy (DCM) is a diabetes mellitus-sparked pathophysiological condition that can result in heart failure (Dillmann, 2019). DCM is mainly characterized by diastolic dysfunction, left ventricular hypertrophy, cardiac stiffness, and myocardial fibrosis in the absence of hypertension or coronary artery disease (Lourenco et al., 2018; Haye et al., 2020). Endothelial cells (ECs) are quiescent under healthy conditions and important to maintain vascular homeostasis and endothelial dysfunction may be the primary mechanism involved in the pathogenesis of DCM (Knapp et al., 2019). ECs can obtain mesenchymal phenotypes and express typical markers of myofibroblastic differentiation, and downregulate the expression of EC markers through a recognized process of endothelial-mesenchymal transition (EndMT; Pardali et al., 2017; Lovisa et al., 2020). Of note, EndMT contributes significantly to myocardial fibrosis, and prevention of EndMT may be a promising therapeutic strategy for DCM (Medici et al., 2011; Zhu et al., 2018; Wang et al., 2020a). However, the mechanisms of EndMT are not fully understood and no treatment to prevent or reverse the underlying molecular changes exists at this time.

Except environmental factors, high fat diets and tobacco smoke, DCM is also influenced by DNA methylation, histone modification, and microRNA (miRNA) expression (Knapp et al., 2019). A number of miRNAs are involved in the pathogenesis of DCM *via* different signaling pathways (Bugger and Abel, 2014; Zhang et al., 2019). miR-195, a vital member of miR-15 family, exerts great influences on cell proliferation, differentiation, and apoptosis (Katsuda and Ochiya, 2012). miR-195 is related to cardiomyocyte apoptosis when upregulated (Evangelista et al., 2019). Essentially, miR-195 is a representative cardio-miR that is upregulated in human heart failure (Katoh, 2014) and is related to insulin secretion in diabetes (Chakraborty et al., 2014). Additionally, the potential role of miR-195-5p in high-glucose (HG)-treated cardiomyocytes has been documented (Shi et al., 2020). Importantly, miR-195 promotes myocardial fibrosis in rats with myocardial infarction *via* targeting transforming growth factor beta (TGF-β)/Smad (Wang et al., 2020b). Increased collagen deposition responsible for fibrosis may be related to an increased expression of TGF-β (Evangelista et al., 2019). Activation of the profibrotic transforming growth factor beta 1 (TGF-β1)/Smad pathway increases myocardial collagen, fibronectin content, and interstitial fibrosis in concert with impaired insulin signaling (DeMarco et al., 2014; Jia et al., 2015). TGF-β by inducing EndMT further contributes to fibrosis development (van Meeteren and Ten Dijke, 2012; Sanchez-Duffhues et al., 2018). Activated TGF-β further leads to EndMT and cardiac fibrosis, eventually inducing DCM through the smad-dependent pathways (Tang et al., 2007; Cao et al., 2014; Yue et al., 2017). However, little is known about the mechanism of miR-195-5p in DCM. Consequently, we performed a series of histological and molecular experiments to identify the mechanism of miR-195-5p in DCM, with the purpose to provide some novel therapies against DCM.

## Materials and Methods

### Ethics Statement

This study was performed with the approval of the Clinical Ethical Committee of Meishan People’s Hospital. All animal experiments were carried out in line with the guidelines for laboratory animal care and use of the National Institutes of Health, and were approved by the animal care and use committee of Meishan People’s Hospital. All experimental procedures were implemented on the ethical guidelines for the study of experimental pain in conscious animals.

### Establishment of DCM Rat Model

Male Sprague Dawley rats (180–220g) purchased from the animal center of the Second Affiliated Hospital of Harbin Medical University raised in a room under 12-h dark/light cycles (lights on at 7:00a.m.) with a constant humidity of 55±5%. The rats were randomly allocated into four groups: control group, DCM group, DCM+antagomir NC (negative control) group, and DCM+miR-195-5p antagomir group. DCM rats were fed with a high-fat diet for 8weeks, including 10% refining lard, 20% sucrose, 8% custard powder, 2% cholesterin, and 60% normal chow. Then, the rats were intraperitoneally injected with 30mg/kg/day streptozocin (Sigma-Aldrich, Merck KGaA, Darmstadt, Germany) for 3days. The fasting blood glucose level was detected after 72h. The rats with fasting blood glucose >16.7mmol/L was considered as successful DCM models (Wang et al., 2019). Control rats received a normal diet. For DCM+antagomir NC group and DM+miR-195-5p antagomir group, DCM rats were injected with antagomir NC or miR-195-5p antagomir (40mg/kg/day) *via* caudal vein for 3 consecutive days (Wang et al., 2020c). After 4weeks of intravenous injection, the rats were euthanized with excessive pentobarbital sodium (100mg/kg, i.p.). There were 12 rats in each group, with six for tissue sectioning and six for protein RNA extraction. The modeling process was shown in Supplementary Figure 1. Antagomir NC and miR-195-5p antagomir were purchased from Shanghai GenePharma Co, Ltd (Shanghai, China).

### Assessment of Cardiac Function

Vevo 770 imaging system (VisualSonics, Toronto, Canada) was adopted to testify the cardiac function of rats. Echocardiographic parameters included the ratio of diastolic transmitral flow velocity (E/A) in early (E) and late atrial (A), the circumferential strain rates of early diastolic (SRe) and late diastolic (SRa) obtained from parasternal short axis gray-scale images, left ventricular anterior wall (LVAW), left ventricular posterior wall thickness (LVPW), left ventricular ejection fraction (LVEF), and fractional shortening (FS).

### Masson Staining

After euthanasia, the heart was removed. Myocardial tissue samples were fixed in 4% paraformaldehyde and embedded in paraffin after dehydration. Next, paraffined samples were cut into 5μm sections using tissue processing equipment. Then, the sections were dewaxed according to the standard procedure and the left ventricular myocardial tissue was used for Masson staining. The sections were treated with 1% hydrochloric acid for 5min, dyed with basic fuchsin solution for 3min, treated with 1% phosphomolybdate for 1min, dyed with 2% aniline blue solution for 2min, and dehydrated with 95% ethanol. The results were observed under the microscope (Olympus, Tokyo, Japan). The collagen fibers were dyed blue, the cytoplasm was red, and the nucleus was black (Xu et al., 2020). Quantitative assessment was performed in randomly selected areas (200×).

### Sirius Red Staining

Left ventricular myocardial tissue sections were stained with Sirius red using a kit (ab150681, Abcam, Cambridge, CA, United States). The procedures of dewaxing, incubation with staining solution and dehydration were simply followed a recent study (Kraus et al., 2020). Photographs were captured with an optical microscope (Olympus; 200 ×). The red area of collagen deposition was counted by Image Pro Plus 6.0 (Media Cybernetics, Bethesda, MD, United States; Zeng et al., 2020).

### Immunohistochemistry

After dewaxing and rehydration of paraffined sections, the endogenous peroxidase activity was quenched by incubation in 0.3% H_2_O_2_ at 37°C for 30min. After washing with phosphate-buffered saline (PBS), the tissue sections were boiled at 100°C in 10mmol/L citrate buffer (pH 6.0) for 30min. Once cooled to room temperature, the sections were sealed with 5% normal goat serum at 37°C for 1h, followed by incubation with antibodies against collagen I (ab254113, 1:100), collagen III (ab7778, 1:100), fibronectin (ab268020, 1:2000), or MMP-9 (ab76003, 1:100) at 4°C overnight. After three times of washing with PBS, the sections were incubated with the secondary antibody IgG (ab205718; 1:2,000) at 37°C for 1h. After washing with PBS three times, the sections were incubated with horseradish peroxidase bound streptomyces avidin (1:1,000) at 37°C for 45min. The newly prepared 2,4-diaminobutyric acid was added for color development. All sections were counterstained with hematoxylin and analyzed under an Olympus BX51 microscope.

### Reverse Transcription Quantitative PCR

Total RNA was extracted using the RNeasy Mini Kit (Qiagen, Valencia, CA, United States). The reverse transcription kit (RR047A, Takara, Tokyo, Japan) was used to reverse RNA into cDNA. The reverse transcription quantitative PCR (RT-qPCR) detection was performed using the SYBR® Premix Ex Taq™ II (Perfect Real Time) kit (DRR081, Takara) on a PCR real-time system (ABI 7500, ABI, Carlsbad, CA, United States). Each sample was provided with three duplicated wells. The primers were synthesized by Shanghai Biotechnology (Supplementary Table 1). GAPDH or U6 served as internal reference. Gene expression was calculated based on the 2^-ΔΔCt^ method.

### Western Blot Analysis

The tissues or cells were lysed with enhanced RIPA lysate containing protease inhibitor (Boster Biological Technology Co., Ltd., Wuhan, Hubei, China) and the protein concentration was determined using the bicinchoninic acid protein assay kit (Boster). The extracted protein was subjected to electrophoresis separation and then transferred to polyvinylidene fluoride membranes. After that, the membranes were blocked with 5% bovine serum albumin for 2h for blocking nonspecific binding, and incubated with primary antibodies against rabbit anti collagen I (ab254113, 1:1,000), collagen III (ab7778, 1:5,000), fibronectin (ab268020, 1:1,000), MMP-9 (ab76003, 1:1,000), CD31 (ab222783, 1:2,000), α-smooth muscle actin (α-SMA; ab108424, 1:1,000), vascular endothelial cadherin (VE-cadherin; ab231227, 1:1,000), vimentin (ab92547, 1:1,000), snail (ab82846, 1:500), twist (ab49254, 1:500), TGF-β1 (ab215715, 1:1,000), smad2/3 (ab202445, 1:1,000), p-smad2/3 (ab272332, 1:1,000), smad7 (ab272928, 1:2,000), and β-actin (ab8226, 1:2,500) overnight at 4°C. Afterward, the membranes underwent a 1-h incubation with the goat anti-rabbit secondary antibody HRP-labeled IgG (ab205718, 1:2,000). Enhanced chemiluminescence reagent (EMD Millipore, Billerica, MA, United States) was used for development. The Image Pro Plus 6.0 (Media Cybernetics, San Diego, CA, United States) was employed for gray value quantification.

### Culture and Treatment of HUVECs

Human umbilical vein endothelial cells (HUVECs; ATCC) were cultured with CC-3162 EGM-2 Bulletkit (Lonza, MD, United States). According to the instructions of Lipofectamine 2000 (Invitrogen, Carlsbad, CA, United States) and opti-MEM reduced serum medium (GIBCO life technologies, Grand Island, NY, United States), the cells cultured for 24h in 12-well plates were treated with miR-195-5p inhibitor, miR-195-5p mimic, si-smad7, or corresponding controls (GenePharma). After 6h, opti-MEM reduced serum medium was replaced by complete cell culture medium with high concentration of glucose (HG: 25mmol/L, D-glucose, G5500, Sigma-Aldrich) and negative control (NG: 25mmol/L, L-glucose, G8644, Sigma-Aldrich) for 48h (Li et al., 2020). In order to further verify the regulatory effect of TGF-β1/smads pathway, HUVECs were stimulated using 3μmol/L TGF-β1R-I inhibitor LY-364947 (LY, # 54678S, CST, Beverly, MA, United States) for 24h (Che et al., 2020). The cell treatment process was shown in Supplementary Figure 2.

### Immunocytochemistry and Immunofluorescence Staining

Myocardial tissue and HUVECs were fixed with 4% paraformaldehyde and treated with 0.5% Triton X-100. Then they were blocked with 5% bovine serum albumin and incubated with anti-α-SMA (#19245,1:500, CST, Shanghai, China) and CD31 (ab24590, 1:1,000, Abcam) or rabbit anti-VE-cadherin (ab33168, 1:1,000, Abcam), and vimentin (ab8978, 1:1,000, Abcam) at 4°C. The next day, the cells were incubated with the corresponding fluorescent antibodies Alexa Fluor 488-labeled goat anti-rabbit IgG (ab150077, 1:1,000, Abcam) and Alexa Fluor 647-labeled goat anti-mouse IgG (ab150115, 1:200, Abcam). Finally, the nuclei were stained with 4',6-diamidino-2-phenylindole and cells were observed using confocal microscopy (Olympus, Tokyo, Japan).

### Dual-Luciferase Reporter Gene Assay

The synthetic Smad7 3'UTR gene fragment Smad7 wild type (WT) and Smad7 mutant (MUT) with binding site mutation were constructed into pMIR reporter plasmid (Beijing Huayueyang Biotechnology, Beijing, China). Luciferase reporter plasmids (smad7-WT and MUT) were cotransfected with miR-195-5p into HEK293T cells (Shanghai Beinuo Biotechnology, Shanghai, China). At 48h post transfection, the cells were lysed. Luciferase assay kit (K801-200; Biovision, Mountain View, CA, United States) was used for detection of luciferase activity.

### RNA Immunoprecipitation

The binding of miR-195-5p with smad7 was detected by RNA immunoprecipitation (RIP) Kit (Millipore, Bedford, MA, United States). The cells in each group were washed with precooled PBS and the supernatant was discarded. The cells were lysed with the same volume of RIPA lysate (P0013b, Beyotime Biotechnology Co., Ltd., Shanghai, China) in ice bath for 5min and centrifuged at 4°C for 10min to obtain the supernatant. The cell extract was incubated with the antibody for coprecipitation. The samples were detached using proteinase K and RNA was extracted for detection of miR-195-5p and Smad7 by RT-qPCR. The rabbit anti-AGO2 antibody (1:100, ab32381, Abcam) was mixed for 30min, and rabbit anti human IgG (1:100, ab109489, Abcam) was used as NC.

### Statistical Analysis

All data were processed using SPSS 21.0 (IBM Corp., Armonk, NY, United States). The measurement data were expressed in the form of mean±SD. Firstly, the data were verified to conform to normal distribution and homogeneity of variance. Comparison between two groups was analyzed using the *t*-test. Comparison among groups was analyzed using one-way ANOVA. If the data did not conform to normal distribution or homogeneity of variance, the rank-sum test was employed for the nonparametric analysis. *p*<0.05 was indicative of a statistically significant difference.

## Results

### Inhibition of miR-195-5p Alleviates Cardiac Dysfunction in DCM Rats

**Figure 1:**
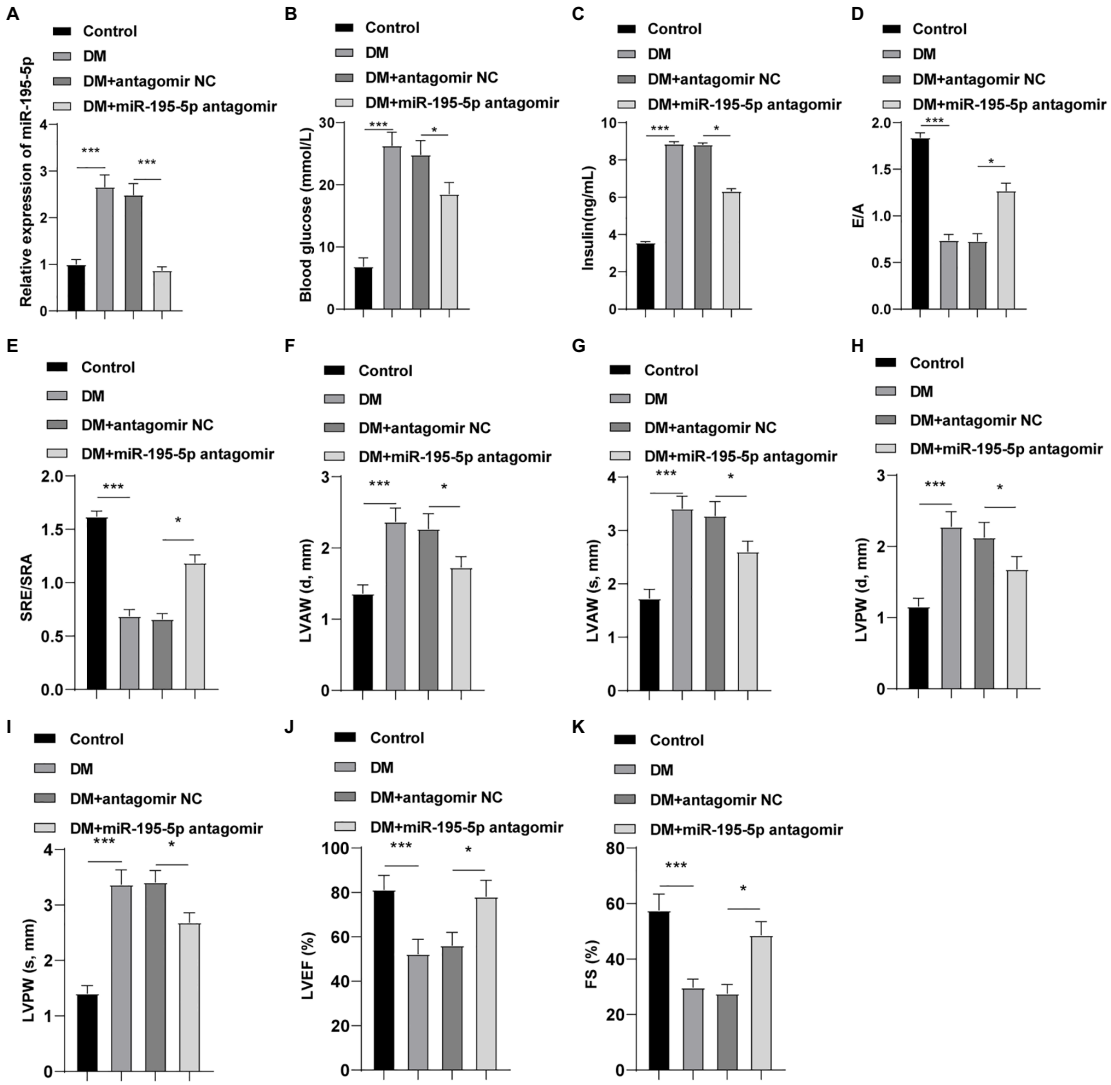
Inhibition of microRNA (miR)-195-5p reduces cardiac dysfunction in diabetic cardiomyopathy (DCM) rats. **(A)** Expression changes of rats in each group were detected using reverse transcription quantitative PCR (RT-qPCR); **(B)** Blood glucose changes of rats in each group; **(C)** Insulin level changes of rats in each group; **(D)** E/A ratio of rats in each group; **(E)** SRe/SRa ratio of rats in each group; **(F)** Relative diastolic left ventricular anterior wall (LVAW) in rats; **(G)** Relative systolic LVAW in rats; **(H)** Relative diastolic left ventricular posterior wall (LVPW) in rats; **(I)** Relative systolic LVPW in rats; **(J)** Relative left ventricular ejection fraction (LVEF) value in rats; and **(K)** Relative fractional shortening (FS) value in rats; N=12 rats in each group; the data were expressed as mean±SD, and analyzed by one-way ANOVA and Tukey’s multiple comparisons test; ^*^p<0.05, ^***^p<0.001. d, diastole; s, systole.

The expression of miR-195-5p was silenced in DCM rats, and the changes of the expression of miR-195-5p after 4weeks of intervention in rats were measured. Compared with the control group, the expression of miR-195-5p in the DCM group was significantly upregulated (*p*<0.05, Figure 1A), while miR-195-5p expression in the DCM+miR-195-5p antagomir group was lower than that in the DCM+antagomir NC group (*p*<0.05, Figure 1A). Subsequently, the changes of blood glucose and insulin level in DCM rats were measured. Compared with the control group, the blood glucose and insulin level were significantly increased in the DCM group, while blood glucose and insulin level in the DCM+miR-195-5p antagomir group were lower than that in the DCM+antagomir NC group (Figures 1B,C). In order to further determine whether miR-195-5p can affect the cardiac function of DCM rats, we collected and counted the relevant indicators of cardiac function. The diastolic function of DCM rats was evaluated by E/A ratio and SRe/SRa ratio. Compared with the control group, E/A ratio and SRe/SRa ratio in the DCM group were significantly decreased, while miR-195-5p antagomir partially reversed the decrease (Figures 1D,E). Furthermore, the diastolic anterior wall thickness (LVAW; d), systolic anterior wall thickness (LVAW; s; Figures 1F,G), diastolic posterior wall thickness (LVPW; d), and systolic posterior wall thickness (LVPW; s; Figures 1H,I) were significantly increased in DCM rats, while the increase of LVAW was attenuated by miR-195-5p antagomir. In addition, LVEF and FS were evidently decreased in DCM rats, while miR-195-5p antagomir partially reduced the decrease of LVEF and FS in DCM rats (Figures 1J,K). Briefly, inhibition of miR-195-5p can reduce cardiac dysfunction in DCM rats.

### Inhibition of miR-195-5p Reduces Myocardial Fibrosis in DCM Rats

**Figure 2:**
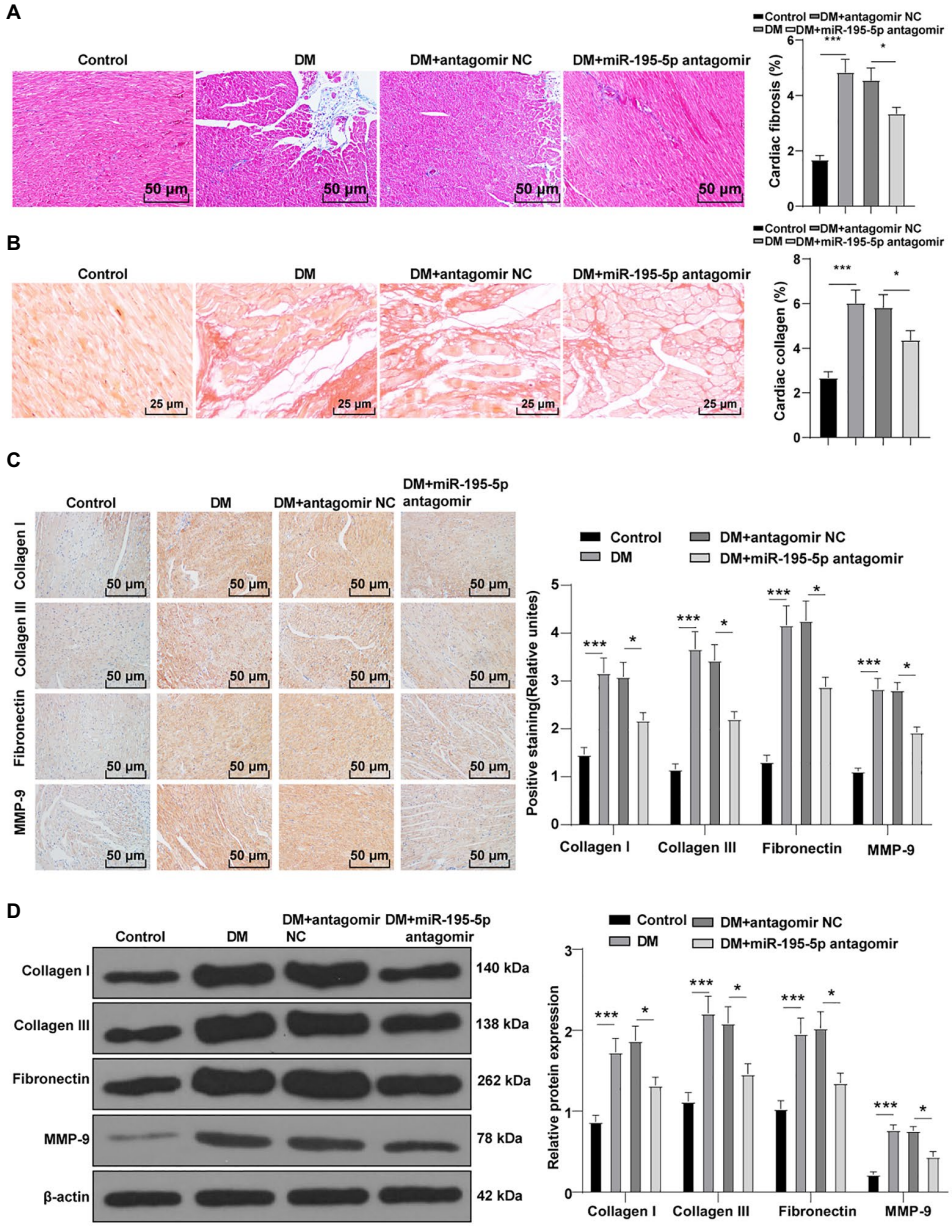
Inhibition of miR-195-5p reduces myocardial fibrosis in DCM rats. **(A)** Masson staining, scale bar=50μm, 200 ×; **(B)** Sirius red standing, scale bar=50μm, 200 ×; **(C)**: Immunohistochemistry was adopted to detect the expression of collagen I, collagen III, fibronectin, and MMP-9; and **(D)** Western blot was used to detect the levels of collagen I, collagen III, fibronectin, and MMP-9. N=12 rats in each group; the data were expressed as mean±SD, and analyzed by one-way ANOVA and Tukey’s multiple comparisons test; ^*^p<0.05, ^***^p<0.001.

To identify the effect of miR-195-5p on myocardial fibrosis in DCM rats, Masson staining and Sirius red standing were performed on myocardial tissue. Compared with the control group, myocardial fibrosis and collagen deposition were increased in the DCM group, but significantly decreased after miR-195-5p silencing (*p*<0.05; Figures 2A,B). The expression of molecular markers of fibrosis collagen I, collagen III, fibronectin, and MMP-9 (Li et al., 2020) in myocardial tissue was detected by immunohistochemistry. Compared with the control group, the expression of these fibrosis molecular markers was significantly increased in the DCM group, but decreased after miR-195-5p silencing (*p*<0.05; Figure 2C). Western blot results were consistent with immunohistochemistry results. Silencing miR-195-5p significantly inhibited the protein levels of collagen I, collagen III, fibronectin, and MMP-9 (*p*<0.05; Figure 2D). In short, inhibition of miR-195-5p can reduce myocardial fibrosis in DCM rats.

### Inhibition of miR-195-5p Reduces Myocardial EndMT in DCM Rats

**Figure 3:**
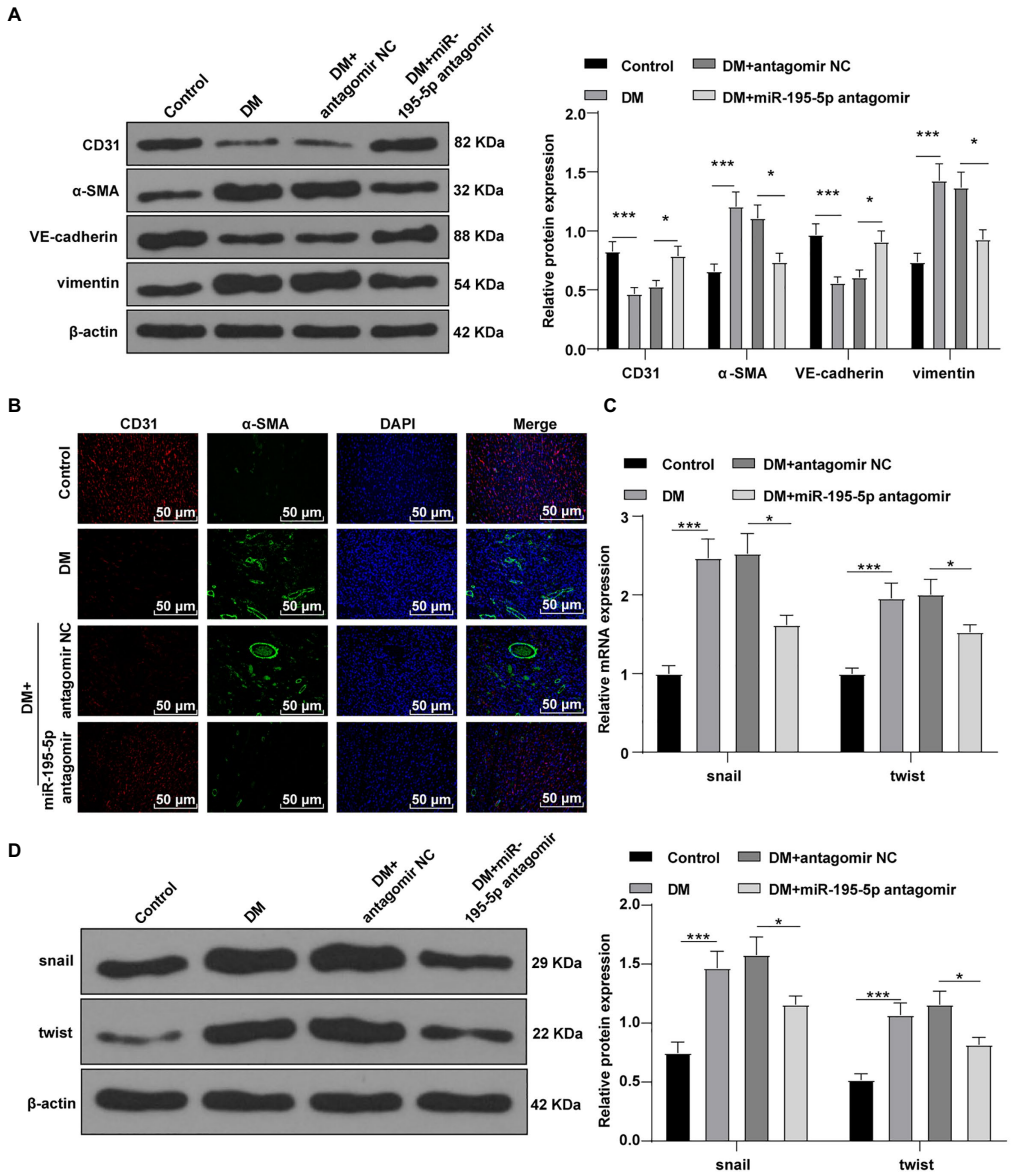
Inhibition of miR-195-5p can reduce the endothelial mesenchymal transition (EndMT) in DCM rats. **(A)** Western blot was used to detect the levels of CD31, α-SMA, VE-cadherin, and vimentin; **(B)** Immunofluorescence was used to detect the expression of CD31 and α-SMA in myocardial tissue, scale bar=25μm, 400 ×; **(C)** RT-qPCR was used to detect the expression of snail and twist; and **(D)** Western blot was used to detect the levels of snail and twist; N=12 rats in each group; the data were expressed as mean±SD, and analyzed by one-way ANOVA and Tukey’s multiple comparisons test; ^*^p<0.05, ^***^p<0.001.

Endothelial mesenchymal transition is the result of HG-induced endothelial damage, which is related to the pathology of myocardial fibrosis in DCM (Zheng et al., 2019; Li et al., 2020). Then we detected the expression of endothelial markers (CD31, VE-cadherin) and fibrosis markers (α-SMA, vimentin; Zheng et al., 2019) by Western blot to study the effect of miR-195-5p on EndMT. Compared with the control rats, the levels of CD31 and VE-cadherin were significantly decreased, the expressions of α-SMA and vimentin were increased in DCM rats, while CD31 and VE-cadherin were significantly promoted by silencing miR-195-5p, and α-SMA and vimentin were inhibited (all *p*<0.05; Figure 3A). Immunofluorescence assay also showed that the endothelial marker CD31 was decreased, while the expression of fibrosis marker α-SMA was increased in DCM rats; while silencing miR-195-5p averted these outcomes (Figure 3B). RT-PCR and Western blot were utilized to examine the molecular markers of EndMT (snail and twist) in myocardial tissue. Compared with the control group, the expression of snail and twist was evidently increased in the DCM group, while miR-195-5p silencing notably inhibited the expression of snail and twist (*p*<0.05; Figures 3C,D). In brief, inhibition of miR-195-5p can reduce the EndMT in DCM rats.

### Inhibition of miR-195-5p Prevents on HG-Induced EndMT

**Figure 4:**
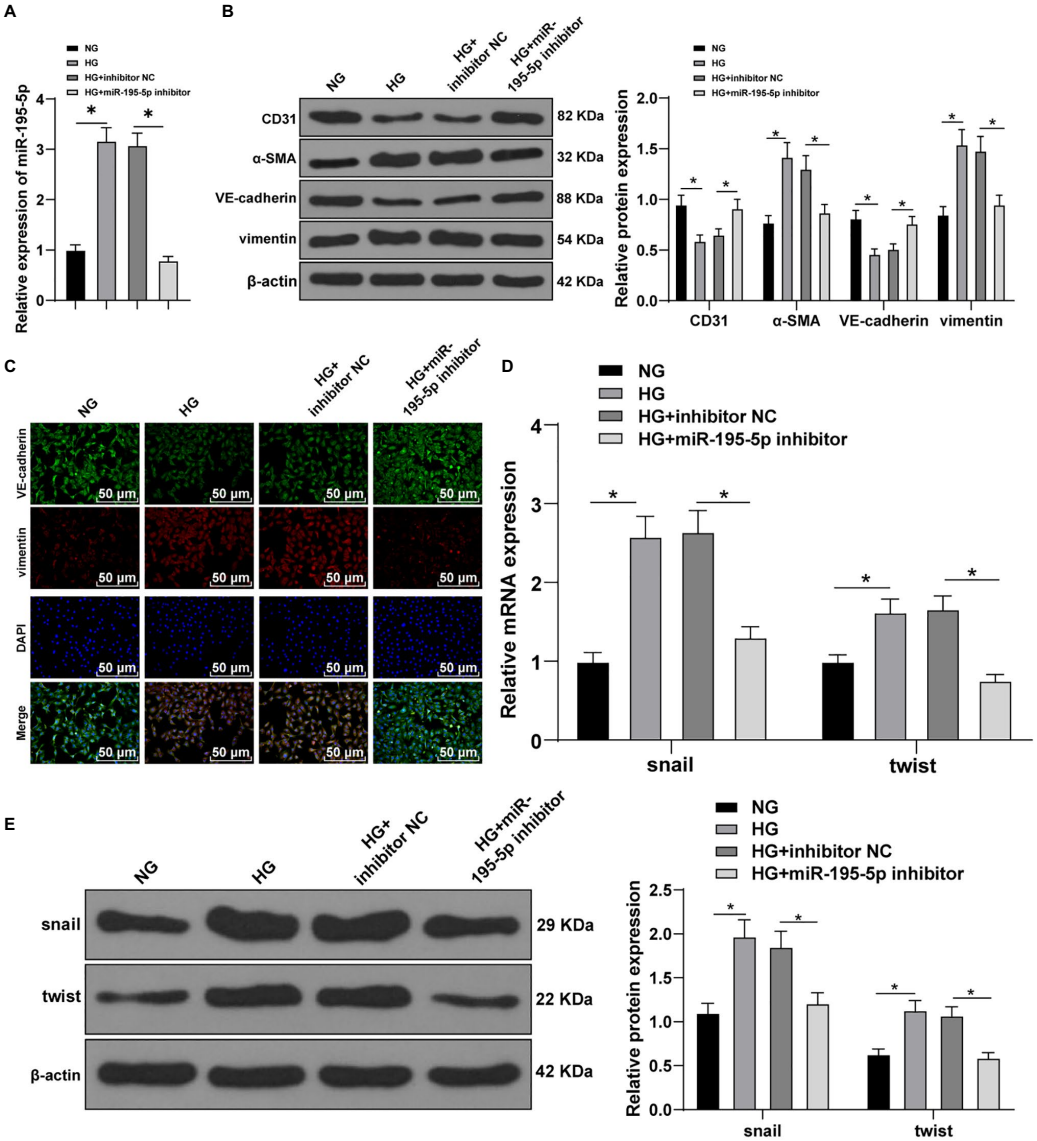
Inhibition of miR-195-5p attenuates high glucose (HG)-induced EndMT in human umbilical vein endothelial cells (HUVECs). **(A)** RT-qPCR was used to detect the expression of miR-195-5p; **(B)** Western blot was used to detect the levels of CD31, α-SMA, VE-cadherin, and vimentin; **(C)** Immunofluorescence was used to detect the expression of VE-cadherin and vimentin in endothelial cells (scale bar=100μm, 100 ×); **(D)** RT-qPCR was used to detect the expression of snail and twist; and **(E)** Western blot was used to detect the levels of snail and twist; the cell experiment was repeated three times. The data were expressed as mean±SD, and analyzed by one-way ANOVA and Tukey’s multiple comparisons test; ^*^p<0.05.

Next, HUVECs were exposed to HG to establish an *in vitro* model to further verify the effect of miR-195-5p on EndMT. RT-qPCR demonstrated that miR-195-5p in the HG group was higher than that in the NG group (*p*<0.05; Figure 4A). Then miR-195-5p inhibitor was used to silence miR-195-5p in endothelial cells. RT-qPCR results showed that compared with the HG+inhibitor NC group, the expression of miR-195-5p in the HG+miR-195-5p inhibitor group was lowered (*p*<0.05; Figure 4A), suggesting that miR-195-5p inhibitor successfully silenced miR-195-5p expression in endothelial cells. Subsequently, the expression of CD31, α-SMA, VE-cadherin, and vimentin was detected by Western blot and immunofluorescence. Compared with the NG group, the levels of CD31 and VE-cadherin in the HG group were obviously decreased and α-SMA and vimentin was increased, while silencing miR-195-5p reversed these protein levels (all *p*<0.05; Figure 4B). The results of immunofluorescence detection were consistent with the trend of Western blot. Silencing miR-195-5p significantly promoted the level of VE-cadehrin and inhibited and vimentin level (Figure 4C). In addition, RT-qPCR and Western blot detected the molecular markers (snail and twist) of EndMT. Compared with the NG group, the expressions of snail and twist were elevated in the HG group, but significantly inhibited after miR-195-5p silencing (*p*<0.05; Figures 4D,E). Altogether, inhibition of miR-195-5p attenuates HG-induced EndMT in HUVECs.

### Inhibition of miR-195-5p Prevents EndMT by Inhibiting the TGF-β1/Smads Pathway

**Figure 5:**
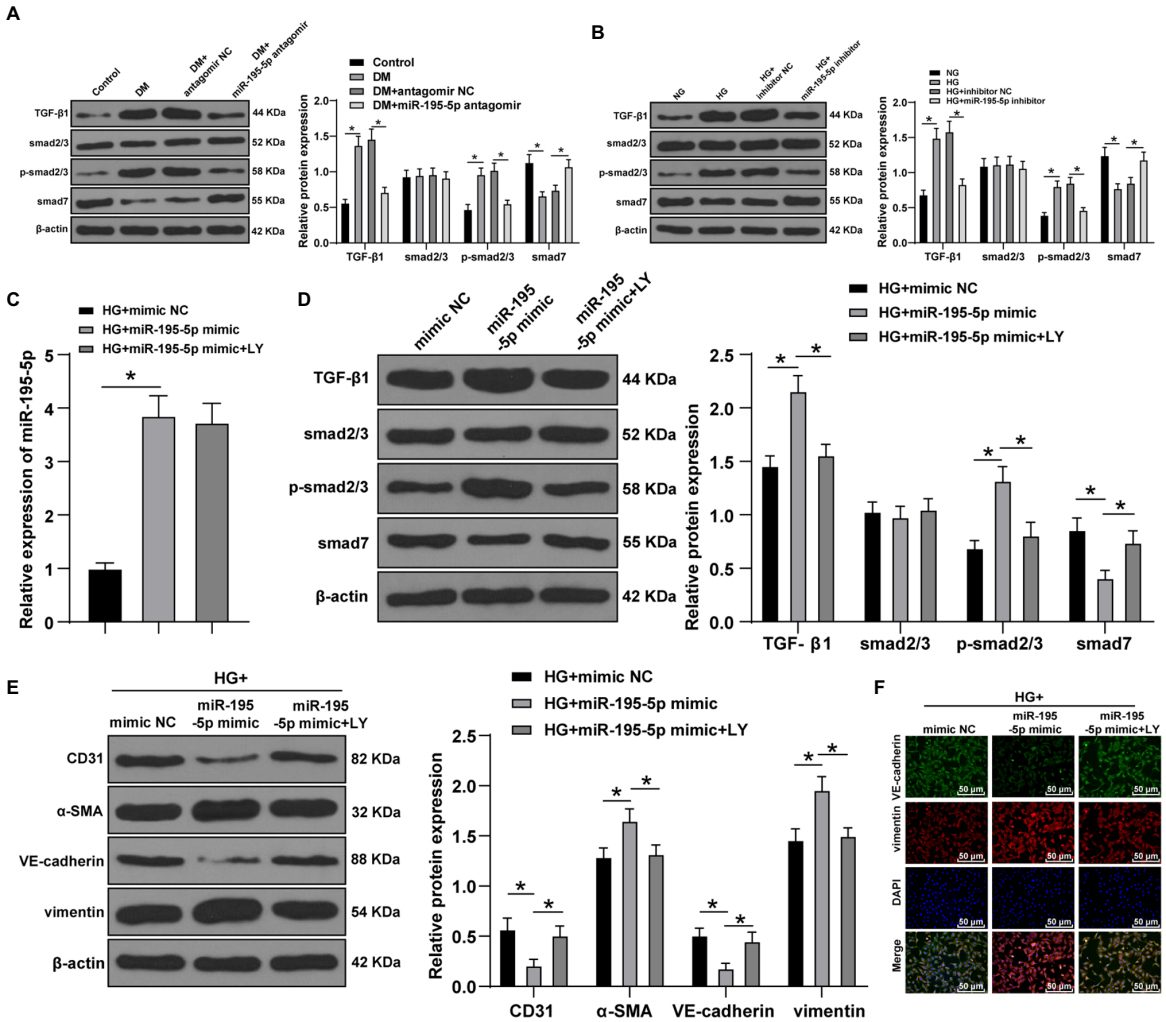
Inhibition of miR-195-5p inhibits EndMT by inhibiting transforming growth factor beta 1 (TGF-β1)-smads pathway. **(A)** Western blot was used to detect the levels of TGF-β1, Smad2/3, p-Smad2/3, and smad7 in myocardial tissue; **(B)** Western blot was used to detect the levels of TGF-β1, Smad2/3, p-Smad2/3, and smad7 in endothelial cells; **(C)** RT-qPCR was used to detect the expression of miR-195-5p in endothelial cells; **(D)** Western blot was used to detect the levels of TGF-β1, Smad2/3, p-Smad2/3, and smad7 after LY-364947 treatment; **(E)** Western blot was used to detect the levels of CD31, α-SMA, VE-cadherin, and vimentin; **(F)** Immunofluorescence was used to detect the expression of VE cadherin and vimentin, scale bar=100μm, 100 ×; the cell experiment was repeated three times. The data were expressed as mean±SD, and analyzed by one-way ANOVA and Tukey’s multiple comparisons test; ^*^p<0.05.

Transforming growth factor beta 1/Smads pathway is the key molecular mechanism of cardiac fibrosis (Che et al., 2020). Western blot was adopted to test the levels of TGF-β1/Smads pathway-related proteins in DCM rats. Compared with control group, the levels of TGF-β1 and p-Smad2/3 in the DCM group were enhanced, while Smad7, an inhibitor of Smad protein family, was significantly decreased; compared with the DCM+antagomir NC group, the DCM+miR-195-5p antagomir group presented significantly decreased expression of TGF-β1 and p-Smad2/3 and increased Smad7 (*p*<0.05; Figure 5A). After silencing miR-195-5p in HG-induced endothelial cells, Western blot showed that HG induced the levels of TGF-β1 and p-Smad2/3 and inhibited Smad7 expression; while silencing miR-195-5p inhibited TGF-β1 and p-Smad2/3 levels and promoted Smad7 (*p*<0.05; Figure 5B).

To verify whether miR-195-5p regulates EndMT through TGF-β1/Smads pathway, LY-364947 (LY) was used to inhibit TGF-β1/Smads pathway. The expression of miR-195-5p in HG+miR-195-5p mimic group was higher than that in the HG+miR NC group (*p*<0.05; Figure 5C); compared with the HG+miR-195-5p mimic group, the miR-195-5p expression in the HG+miR-195-5p mimic+LY group had no significant difference (*p*>0.05; Figure 5C). TGF-β1/Smads pathway related proteins were examined by Western blot. Compared with the HG+miR NC group, the levels of TGF-β1 and p-Smad2/3 in the HG+miR-195-5p mimic group were notably elevated, while Smad7 expression was decreased; compared with the HG+miR-195-5p mimic group, the TGF-β1 and p-Smad2/3 levels in the HG+miR-195-5p mimic+LY group were decreased, while Smad7 expression was increased (*p*<0.05; Figure 5D). The levels of CD31, α-SMA, VE-cadherin, and vimentin were detected by Western blot. Compared with the HG+mimic NC group, the levels of CD31 and VE-cadherin in endothelial cells of the HG+miR-195-5p mimic group were significantly downregulated, and α-SMA and vimentin were increased; compared with the HG+miR-195-5p mimic group, the HG+miR-195-5p mimic+LY group had elevated levels of CD31 and VE-cadherin, and inhibited levels of α-SMA and vimentin (all *p*<0.05; Figure 5E). Meanwhile, the results of immunofluorescence were similar to those of Western blot (Figure 5F). We concluded that inhibition of miR-195-5p inhibits EndMT by inhibiting TGF-β1-smads pathway.

### miR-195-5p Targets Smad7

**Figure 6:**
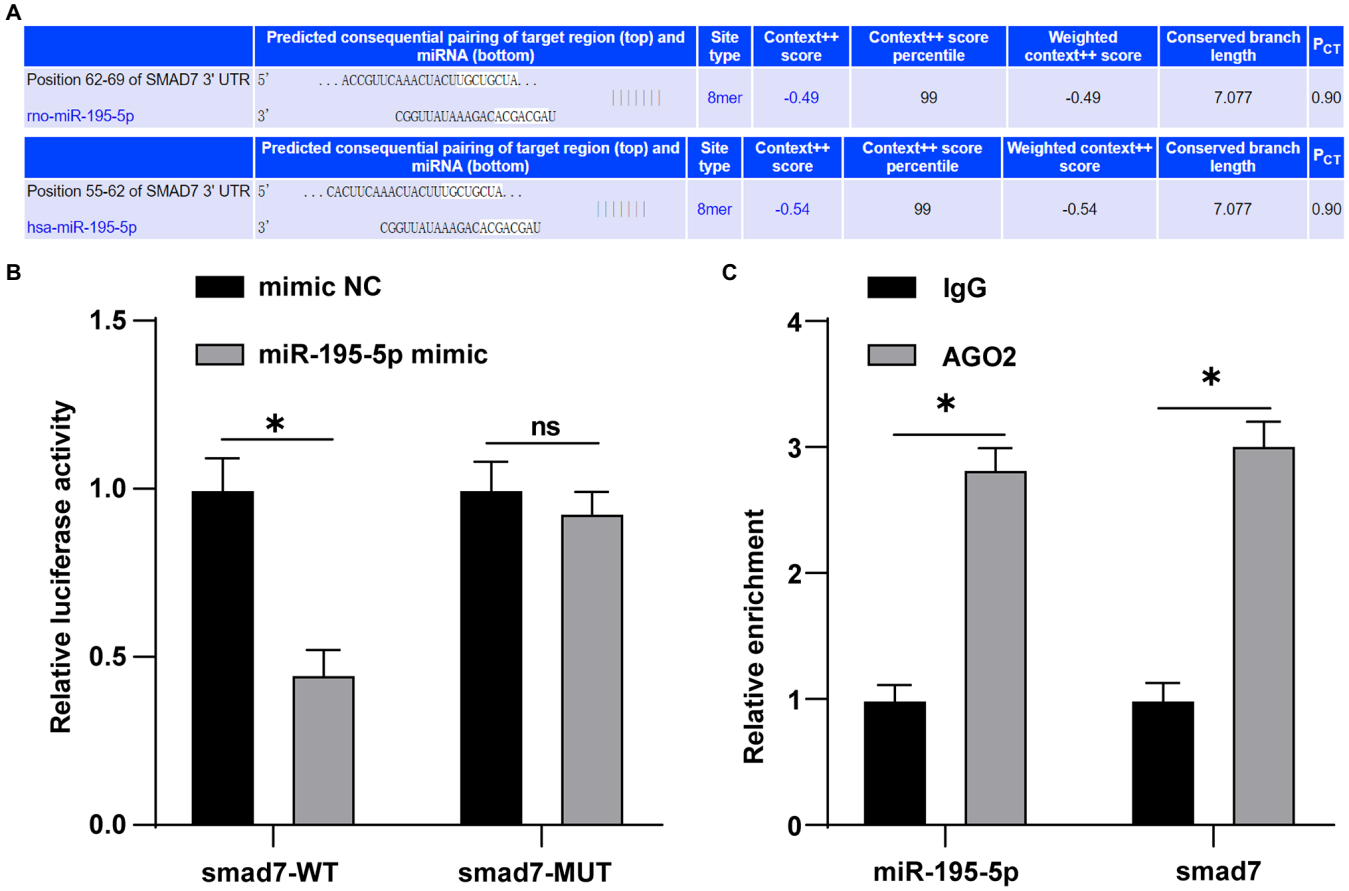
miR-195-5p can bind to smad7. **(A)** TargetScan (http://www.targetscan.org/vert_71/) predicted the binding of miR-195-5 and smad7; **(B)** Dual-luciferase assay showed that miR-195-5 could bind smad7; and **(C)** RNA immunoprecipitation (RIP) assay showed that miR-195-5 could bind to smad7; the cell experiment was repeated three times. The data were expressed as mean±SD, and analyzed by independent *t* test; ^*^p<0.05.

miR-195-5p promotes the proliferation and migration of pulmonary artery smooth muscle cells by targeting smad7 in pulmonary hypertension (Zeng et al., 2018). Whether smad7 is still the target gene of miR-195-5p in endothelial cells is still unclear. Therefore, the TargetScan was used to predict the binding of miR-195-5p and smad7. The results showed that miR-195-5p had binding sites in 3'UTR of smad7 in both rats and humans (Figure 6A). Dual-luciferase assay showed that miR-195-5p mimic significantly inhibited the luciferase activity of smad7-WT (*p*<0.05; Figure 6B), but had no significant effect on smad7-MUT (*p*>0.05; Figure 6B), suggesting that miR-195-5p can bind to smad7. RIP results showed that compared with IgG, AGO2 significantly combined with miR-195-5p and smad7 (*p*<0.05; Figure 6C).

### Silencing miR-195-5p Inhibits EndMT by Promoting Smad7

**Figure 7:**
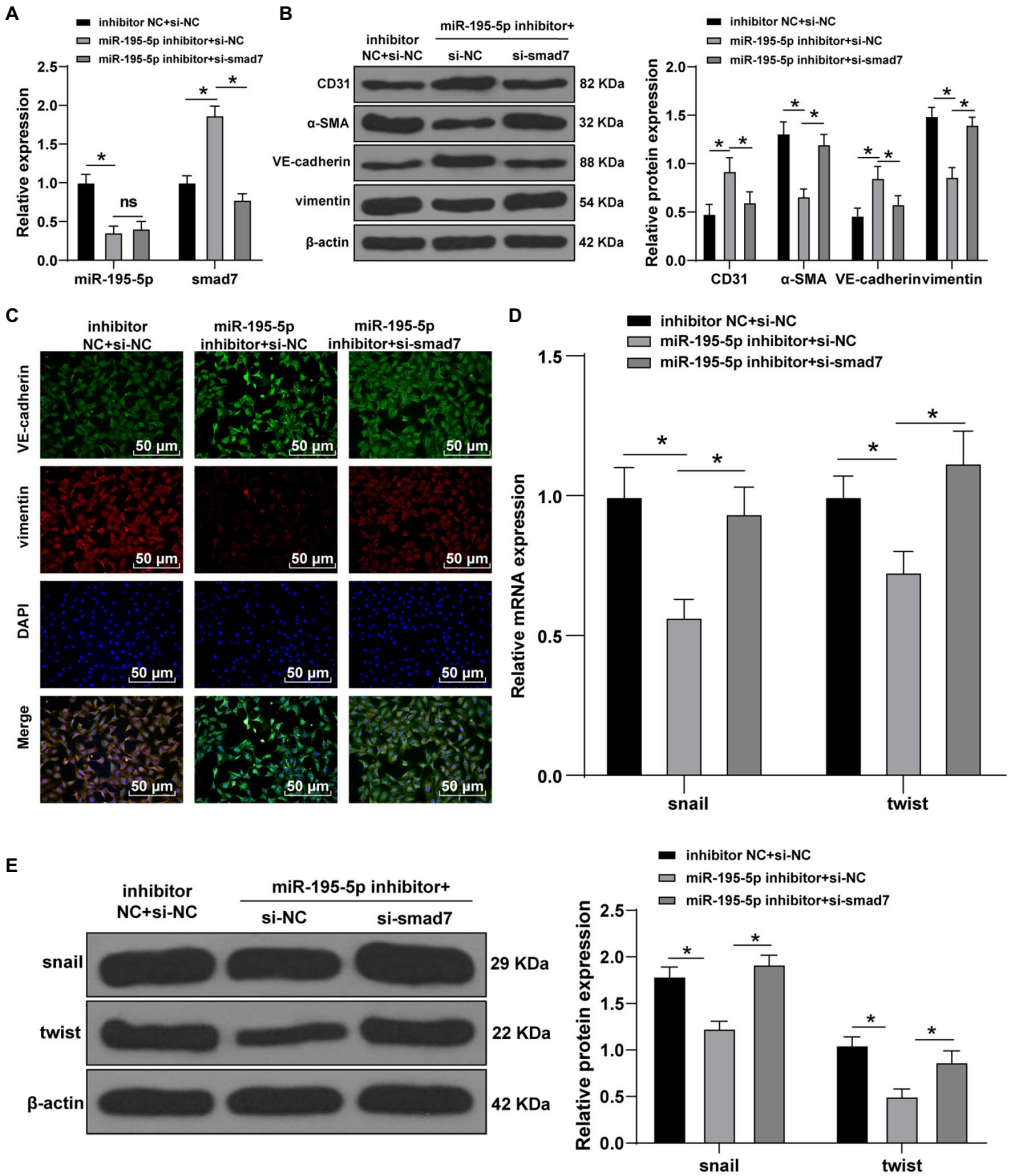
Silencing miR-195-5p inhibits EndMT by promoting smad7 expression. **(A)** The expression of miR-195-5p and smad7 was detected by RT-qPCR; **(B)** The levels of CD31, α-SMA, VE-cadherin, and vimentin was detected by Western blot; **(C)** The expression of VE-cadherin and vimentin was detected by immunofluorescence, scale bar=100μm, 100 ×; **(D)** The expression of snail and twist was detected by RT-qPCR; and **(E)** Western blot was used to detect the levels of snail and twist; the cell experiment was repeated three times. The data were expressed as mean±SD, and analyzed by one-way ANOVA and Tukey’s multiple comparisons test; ^*^p<0.05.

Finally, we silenced miR-195-5p and smad7 simultaneously in ECs cultured in HG conditions. First, the expression of miR-195-5p and smad7 was detected by RT-qPCR. The miR-195-5p was decreased and smad7 was significantly increased after silencing miR-195-5p alone; however, silencing miR-195-5p and smad7 together could reverse the promotion effect of miR-195-5p inhibitor on Smad7 (*p*<0.05; Figure 7A). Western blot and immunofluorescence analysis showed that the levels of VE-cadherin was increased and vimentin was decreased after miR-195-5p was silenced alone, while silencing miR-195-5p and smad7 together partially reversed the effect of miR-195-5p silencing alone (Figures 7B,C). Moreover, the levels of snail and twist were significantly decreased after miR-195-5p was silenced alone, which were partially reversed by silencing miR-195-5p and smad7 (*p*<0.05; Figures 7D,E). Conjointly, silencing miR-195-5p inhibits EndMT by promoting smad7 expression.

## Discussion

Diabetic cardiomyopathy is usually asymptomatic in the early stage of its evolution. One of the earliest manifestations of DCM is LV hypertrophy and/or decreased LV compliance (Jia et al., 2016). EndMT is essential in DCM development (Melendez-Ramirez et al., 2010). Modulating miRNA expression has been identified to reverse histologic and functional measures of DCM in animal models (Quiat and Olson, 2013; Rodino-Klapac, 2013). This study elucidated the mechanism of miR-195-5p in EndMT and myocardial fibrosis in DCM.

A previous miR microarray analysis demonstrates that miR-195 is upregulated in DCM (Diao et al., 2011). Consistently, miR-195-5p expression in DCM rats and HG-induced ECs was significantly increased in the present study. Cardiac miR-195 is upregulated during cardiac hypertrophy, and results in pathological cardiac growth and heart failure (van Rooij et al., 2006). High glucose levels lead to multiple biochemical modifications in ECs in DCM (Knapp et al., 2019). Various studies have shown that the expression of miR-195-5p in the serum of patients with gestational diabetes mellitus (GDM) is a key factor in the pathogenesis of GDM, which may be related to insulin resistance regulation (Tagoma et al., 2018; Wang et al., 2020d). When miR-195-5p was silenced in DCM rats, blood glucose and insulin level were significantly lowered, the increase of LVAW and LVPW was attenuated, and the decrease of LVEF and FS partially reduced. miR-195-5p is upregulated in heart failure, and negatively correlated with left ventricular filling pressure (Marques et al., 2016). Cardiac dysfunction in diabetic hearts progresses from LV fibrosis to diastolic dysfunction and eventually systolic dysfunction accompanied by reduced ejection fraction (Jia et al., 2018). Similarly, the miR-195 antagomir has been documented to contribute to damaged myocardial fibers, proliferation and fibrosis in fibrous tissues, significantly increased LVEF and LVFS, and decreased LVDd and LVDs in rats with myocardial infarction (Wang et al., 2020b). These results suggest that inhibition of miR-195-5p can significantly reduce cardiac dysfunction in DCM rats.

Increased myocardial fibrosis contributes to diastolic dysfunction in DCM (Riehle and Bauersachs, 2018). The collagen I, collagen III, and fibronectin are molecular markers of fibrosis (Li et al., 2020). Our results elicited that myocardial fibrosis and collagen deposition were increased in DCM group but clearly decreased after miR-195-5p silencing. Expression of collagen III is improved in vascular smooth muscle cells after transfection with miR-195 mimic (Liang et al., 2017). MMP-9 is closely related to cardiac wound healing and the active MMP-9 can digest collagen, fibronectin, and laminin (Becirovic-Agic et al., 2021). miR-195 antagomir averts fibronectin upregulation in ECs in diabetic retinopathy (Mortuza et al., 2014). miR-195 promotes myocardial fibrosis in myocardial infarction rats (Wang et al., 2020b). In short, inhibition of miR-195-5p can reduce myocardial fibrosis in DCM rats.

Endothelial mesenchymal transition is a cellular transdifferentiation program, in which ECs partially lose their endothelial identity and acquire mesenchymal-like features and thus contribute to cardiac fibrosis (Lovisa et al., 2020; Wang et al., 2020a). The CD31 and VE-cadherin are endothelial markers and α-SMA and vimentin are fibrosis markers (Zheng et al., 2019). As our results demonstrated, CD31 and VE-cadherin were clearly promoted by silencing miR-195-5p, and α-SMA and vimentin were inhibited. Increased activity of α-SMA is vital in the development of cardiac fibrosis (Li et al., 2012). Levels of mesenchymal markers α-SMA, collagen I, and vimentin are elevated in HG-treated HUVECs, whereas endothelial markers CD31 and VE-cadherin are decreased (Liu et al., 2018). The miR-195 mimic elevates α-SMA in liver fibrotic rats (Song et al., 2017). Inhibition of miR-195 suppresses apoptosis in cardiac ECs in DCM mice (Zheng et al., 2015). Our results unraveled that miR-195-5p silencing also notably inhibited the levels of molecular markers of EndMT (snail and twist). There is little information about the role of miR-195-5p in EndMT, indicating the novelty of our current study. In brief, inhibition of miR-195-5p can reduce the EndMT in DCM rats. Furthermore, EndMT is the result of HG-induced endothelial damage (Zheng et al., 2019; Li et al., 2020). Impaired endothelial function is a typical finding in DCM (Paolillo et al., 2019). Next, we exposed HUVECs to HG to establish an *in vitro* model to further verify the effect of miR-195-5p on EndMT, and confirmed that inhibition of miR-195-5p attenuates HG-induced EndMT in HUVECs.

Transforming growth factor beta 1/Smads pathway is a leading mechanism of cardiac fibrosis (Che et al., 2020). Increased TGF-β1/Smad2/3 signaling cascade is involved in cardiac interstitial fibrosis and cardiac left ventricular remodeling (Jia et al., 2015). The levels of TGF-β1 and p-Smad2/3 in DCM group were enhanced, while Smad7 was significantly decreased. miR-195-5p silencing clearly decreased expression of TGF-β1 and p-Smad2/3 and increased Smad7. To verify whether miR-195-5p regulates EndMT through the TGF-β1/Smads pathway, LY-364947 was introduced to inhibit the TGF-β1/Smads pathway. As expected, TGF-β1 and p-Smad2/3 levels were decreased, while Smad7 was increased in the HG+miR-195-5p mimic+LY group, levels of CD31 and VE-cadherin were elevated, and levels of α-SMA and vimentin were inhibited. TGF-β1 regulates fibroblast proliferation and collagen and fibronectin, and reduces degradation of these components through Smad2/3 (Jia et al., 2015). TGF-β, by inducing transcriptional regulators Snai1, Slug, and Twist, elevates mesenchymal marker α-SMA (Medici et al., 2011; Sanchez-Duffhues et al., 2018). In conclusion, inhibition of miR-195-5p impairs EndMT by inhibiting the TGF-β1-smads pathway.

Several studies have supported that miR-195 exerts regulatory effects by targeting smad7 (Chen et al., 2015; Song et al., 2017; Zeng et al., 2018). Smad7, a negative regulator of TGF-β1 signaling is downregulated in cardiac fibrosis in DCM rats (Meng et al., 2019). Consistently, we found that miR-195-5p can bind to smad7. Then, we silenced miR-195-5p and smad7 simultaneously in ECs in HG conditions. Western blot and immunofluorescence showed that the levels of VE-cadherin was increased and vimentin was decreased after miR-195-5p was silenced alone, while silencing miR-195-5p and smad7 together partially reversed the effect of miR-195-5p silencing alone. Moreover, the levels of snail and twist were decreased when miR-195-5p was silenced, which were reversed by concomitant silencing miR-195-5p and Smad7. Lentiviral delivery of smad7 *in vivo* results in an upregulation of CD31 and VE-cadherin and a downregulation of N-cadherin and vimentin, suggesting that EndMT is blocked in the pathological process of heterotopic ossification due to local smad7 overexpression (Zhang et al., 2016). TGF-β1 increases α-SMA protein and decreases smad7 protein (Song et al., 2019). Collectively, silencing miR-195-5p inhibits EndMT by promoting smad7 expression.

In summary, silencing miR-195-5p inhibits the TGF-β1-smads-snail pathway by targeting smad7, thus blocking EndMT and alleviating myocardial fibrosis in DCM. This study targets an essential clinical research topic, and the research design is comprehensive and logical. The results offer new evidence to improve the understanding of the pathogenesis of human DCM and provide a potential therapeutic potential, thus having high clinical relevance. In addition, this study concentrated on that miR-195 targets the smad7 and that miR195 has a crucial role in the development of cardiac fibrosis *via* modulating EndMT, which is the novelty of this study. However, DCM is a complicated process involving multiple mechanisms except EndMT and myocardial fibrosis. A previous study has shown that the decrease of blood glucose may partly play a role in reducing the myocardial collagen deposition in the treatment of diabetes, but it is not clear whether lowering blood glucose can affect myocardial fibrosis and myocardial dysfunction (Tuleta and Frangogiannis, 2021). The development of DCM involves a variety of mechanisms of decisive molecular, cellular and interstitial changes, including myocardial energy substrate imbalance, glucose and lipid toxicity, insulin signal changes, mitochondrial defects, endoplasmic reticulum (ER) stress, intracellular calcium processing disorder, oxidative stress, endothelial dysfunction, advanced glycation end products (AGEs) deposition, and maladaptive immune response. Each of these can lead to structural remodeling and functional defects in diabetic myocardium, including cardiac relaxation, compliance, and contractile impairment (Salvatore et al., 2021), which also confirms that miR-195-5p can affect blood glucose and EndMT in our study. Furthermore, the regulation mechanism of miRNAs is often not a single and linear structure, but a complex and multiple network structure. A single miRNA can often regulate a variety of target genes and thus regulating biological functions. In this study, we observed that inhibition of miR-195-5p could improve cardiac function and the development of cardiac fibrosis. Over-expression of miR-195-5p has a positive effect on breast cancer (Luo et al., 2014). Therefore, in the future research, it is particularly important to deeply analyze other regulatory mechanisms of miR-195-5p in cardiac fibrosis and dysfunction. More researches and clinical trials are needed to clarify the therapeutic approach and to find new targets for patients affected by DCM. EndMT refers to the process that endothelial cells undergo a series of molecular changes to form mesenchymal like cell phenotypes (such as myofibroblasts). miRNAs such as miR-20a, miR-200a, let-7, miR-21, and miR-630 are related to EndMT. This study found that miR-195-5p was also involved in the development of EndMT. The occurrence and development of this complex biological process is not regulated by a single factor. With the continuous analysis of the regulation mechanism of EndMT, it is believed that it can provide new ideas for the treatment of myocardial diseases.

## Data Availability Statement

All the data generated or analyzed during this study are included in this published article.

## Ethics Statement

The animal study was reviewed and approved by Meishan People’s Hospital.

## Author Contributions

HD is the guarantor of integrity of the entire study. HD, CW, and YJ contributed to the study concepts, study design, and definition of intellectual content. JC, KW, HX, and LL contributed to the literature research. HD, LL, KW, and JY contributed to the manuscript preparation, manuscript editing, and review. HD, JC, and LL contributed to the clinical studies. JY, CW, YJ, and HX contributed to the experimental studies and data acquisition. HD, JC, KW, HX, and LL contributed to the data analysis and statistical analysis. All authors contributed to the article and approved the submitted version.

## Conflict of Interest

The authors declare that the research was conducted in the absence of any commercial or financial relationships that could be construed as a potential conflict of interest.

## Publisher’s Note

All claims expressed in this article are solely those of the authors and do not necessarily represent those of their affiliated organizations, or those of the publisher, the editors and the reviewers. Any product that may be evaluated in this article, or claim that may be made by its manufacturer, is not guaranteed or endorsed by the publisher.

## Supplementary Material

Supplementary Figure 1 Rat modeling process.

Supplementary Figure 2 Cell treatment process.
