# Supplementary figures and images for: MicroRNA-195-5p Downregulation Inhibits Endothelial Mesenchymal Transition and Myocardial Fibrosis in Diabetic Cardiomyopathy by Targeting Smad7 and Inhibiting Transforming Growth Factor Beta 1-Smads-Snail Pathway
Source: Front Physiol. 2021 Sep 30;12:709123. doi: 10.3389/fphys.2021.709123 (PMC8514870; doi:10.3389/fphys.2021.709123)

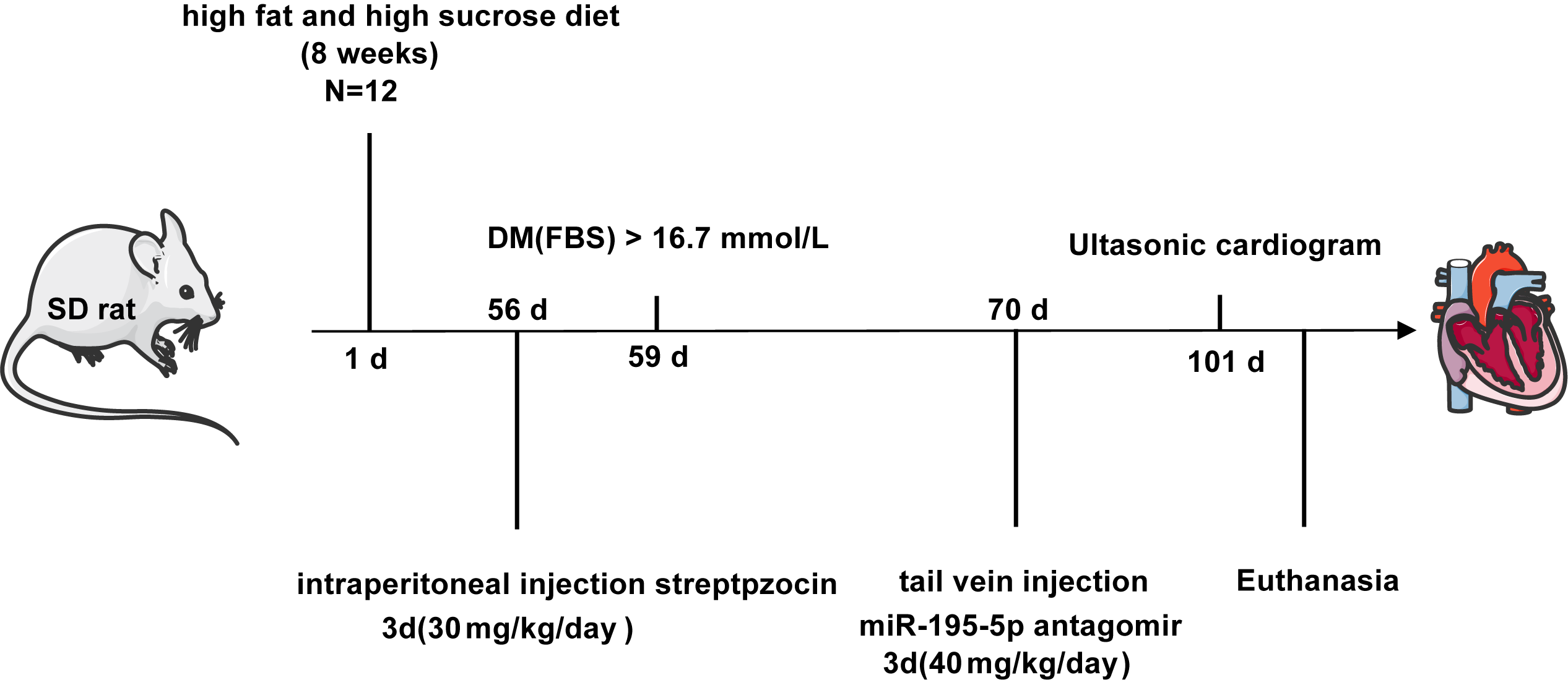

Supplement: Supplementary Figure 1 — Rat modeling process. [file Figure_1.TIFF]

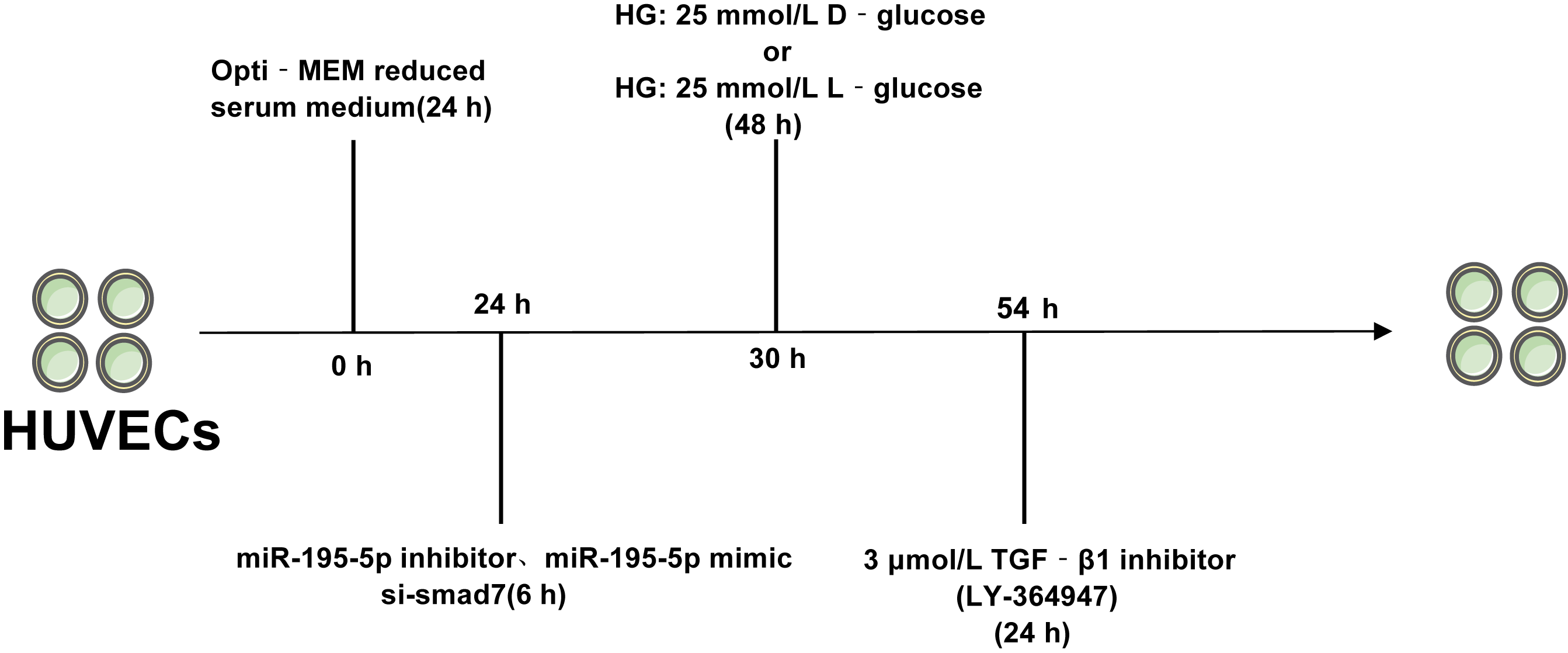

Supplement: Supplementary Figure 2 — Cell treatment process. [file Figure_2.TIFF]
