# Supplementary material for: MicroRNA-195-5p Downregulation Inhibits Endothelial Mesenchymal Transition and Myocardial Fibrosis in Diabetic Cardiomyopathy by Targeting Smad7 and Inhibiting Transforming Growth Factor Beta 1-Smads-Snail Pathway
Source: Front Physiol. 2021 Sep 30;12:709123. doi: 10.3389/fphys.2021.709123 (PMC8514870; doi:10.3389/fphys.2021.709123)
Supplement: Supplementary file 3 [file Table_1.docx]

**Supplementary Table 1** Primer sequence of RT-qPCR

| Gene | Forward Primer (5′-3′) | Reverse Primer (5′-3′) |
| --- | --- | --- |
| miR-195-5p | GGGGTAGCAGCACAGAAAT | TCCAGTGCGTGTCGTGGA |
| snail | CCAAACCCACTCGGATGTGA | TCTTGGTGCTTGTGGAGCAA |
| twist | TCGGACAAGCTGAGCAAGAT | CCAGACGGAGAAGGCGTAG |
| smad7 | GGACGCTGTTGGTACACAAG | GCTGCATAAACTCGTGGTCATTG |
| U6 | GCTTCGGCAGCACATATACTAAAAT | CGCTTCACGAATTTGCGTGTCAT |
| GAPDH | GACATGCCGCCTGGAGAAAC | GACATGCCGCCTGGAGAAAC |
